# The prevalence of oral mucosal lesions and related factors in systemic lupus erythematosus patients

**DOI:** 10.1186/s13075-021-02614-8

**Published:** 2021-09-03

**Authors:** Mayssoun Kudsi, Louei Darjazini Nahas, Rama Alsawah, Ahmad Hamsho, Abdullah Omar

**Affiliations:** 1 Faculty of Medicine, Syrian Private University, Damascus, Syrian Arab Republic; 2 Rheumatology Department, Damascus University, Damascus, Syrian Arab Republic; 3 Otorhinolaryngology Department, Syrian Private University, Damascus, Syrian Arab Republic

**Keywords:** Systemic lupus erythematosus, Oral lesions, Mucosal lesion

## Abstract

**Background:**

Systemic lupus erythematosus (SLE) is a chronic inflammatory multi systematic disease of unknown aetiology. SLE has a wide range of symptoms. The most common symptoms are joint pain, skin rash and fever. Oral lesions in SLE manifest in a variety of forms, such as oral mucosal ulceration, mouth burns, xerostomia and salivary gland diseases, temporomandibular joint disease, periodontal disease, dysgeusia, white lesions, oedema, bleeding and petechiae.

**Objective:**

This study was conducted to evaluate the prevalence of oral mucosal lesions and their related factors in patients with SLE, giving the lack of comprehensive statistical data in Syria and the differences between reported prevalence.

**Patients and methods:**

A cross-sectional study was performed in the Al-Mouassat University Hospital in Damascus. Patients were evaluated appropriating observation, clinical examination, completing questionnaires, studying patient’s medical records and paraclinical laboratory tests if required. Four types of oral lesions were evaluated: ulcer, erythema, white plaque and spots. The diagnosis of these lesions was made according to observation and clinical examination, and the location of each lesion was also recorded. Data were analysed using SPSS version 16.0.

**Result:**

In this study, 42 (70% %) out of 60 patients (38 women and 4 men) had oral lesions, while 18 (30%) had none. The most common areas for the lesions were the buccal mucosa (26.1%) and the lips (14.2%). Of the 42 patients with oral lesions, 12 (27.6%) showed ulcers. There was a significant relationship between the following factors and oral lesions: oral hygiene status, the duration of the disease involvement, frequency of pregnancies, the amount of daily use of corticosteroids without significant difference between dosage groups, and medications used for SLE treatment other than corticosteroids (*p* < 0.008) without mentioned names or dosages. Conversely, age, sex, cigarette smoking and medications other than those used for SLE treatment were not significantly related to the presence of oral lesions (*p* value was greater than 0.05 in all subjects).

## Introduction

Systemic lupus erythematosus is an idiopathic chronic multiple inflammatory disease [1]. It is autoimmune, i.e., the body’s immune system (antibodies) mistakenly attacks its tissues, causing inflammation of multiple organs, especially the heart, lungs, bones, joints, kidneys and skin. Clinical manifestations may vary between patients, but most manifest with musculoskeletal involvement, especially arthritis of the limbs (inflammation of the small and large joints), while others may experience a wide range of symptoms, most commonly joint pain, rash and fever. These symptoms can develop slowly or appear suddenly [2–4]. The disease is characterized by periods of remission and exacerbation, and the period of remission may last several years [5, 6].

The diagnosis of lupus is rather complicated because, as stated, the symptoms vary widely between the patients. In addition to that, the presentation can resemble several other immune diseases. The diagnosis is based on specific criteria that include symptoms, signs and laboratory evaluations, such as positive anti-nuclear antibodies [7].

Oral lesions are common in many patients with systemic lupus erythematosus (SLE) and are considered one of the diagnostic criteria according to the American Society of Arthritis and Rheumatism 1982 [8]. Its prevalence varies according to the type of lupus: 8–45% in patients with systemic lupus, 3–20% in patients with chronic cutaneous lupus and 4–25% in patients with discoid lupus erythematosus [8, 9].

Oral lesions manifest in a variety of forms, such as oral mucosal ulcers, that occur in more than 40% of patients [10], dry mouth, lesions of the salivary gland, temporomandibular disorders (TMDS TMJ), gum lesions, and distortion in paste, white lesions, oedema, gum bleeding and bruising [11–15].

The exocrine glands are also affected, especially the eyes and mouth, as 50–75% of patients may complain of dry mouth as flow rate decreases in the salivary glands of many patients. Lupus may also be associated with secondary Sjogren’s syndrome [16–19].

Drugs used to control the disease, whether for a short or long period, have several side effects on the oral cavity. Steroids, for example, lead to calcifications and fragmentations in the root canals and therefore increase the susceptibility to necrosis. NSAIDs, on the other hand, can lead to gingival bleeding and hypertrophy, which may occur after the use of cyclosporine as well. They also delay alveolar bone resorption. On the other hand, gum health has also been found to improve in some patients taking these medications. Some drugs may also increase the occurrence of oral infections, such as infections with *Candida*, other fungi and viruses [20–25].

This study was conducted to evaluate the prevalence of oral mucosal lesions and their associated factors in patients with systemic lupus. This is due to the abundance of these lesions, in addition to the lack of comprehensive statistical data regarding this issue in Syria.

## Materials and methods

A cross-sectional study was conducted at Al-Mouwasat University Hospital in Damascus from 2012 till 2014 on patients with systemic lupus erythematosus who are either attending outpatient clinics or admitted into the hospital. Collecting data was done by reading the patient’s files, interviewing them for patient history and filling in a form, performing a clinical examination and requesting a laboratory evaluation when necessary.

The disease was diagnosed based on the criteria of the American Society of Arthritis [26].

Only 60 patients, both male and female, were included in the study. This is due to the insufficient number of submitted patients and the exclusion of some for not being able to follow-up. The forms were filled in by taking age, gender, number of pregnancies after illness, smoking, disease duration, the use of medications with their doses and oral health statuses, such as the presence and absence of natural teeth and oral hygiene [27–30].

Patients were divided into groups according to the duration of illness into less than 3 months, between 3 and 6 months, 6–12 months and more than 12 months, based on reading the included studies [31–34].

Patients taking oral steroids were divided according to the dose into the following groups: less than 7.5 mg prednisolone per day, between 7.5 and 60 mg per day and more than 60 mg per day, given that the maintenance dose is 7.5 mg /day and 60 is equivalent to 1 mg/kg per day. Patients were estimated to weigh approximately 60 kg, with no scientific documents or epidemiological studies discussing this issue in Syria.

Accompanying drugs, whether anti-malarial drugs or immunosuppressants, were studied.

The oral injury was evaluated with four manifestations according to the clinical examination: the presence of an ulcer that includes the lining and the deeper layers, erythema, a white plaque with a height of more than 1 cm and white or red spots.

## Statistical study

The statistical study was done using SPSS, version 16.0, which was used to link the independent variables, except for the numerical variables. *p* value was considered statistically significant when less than 0.05.

## Results

In this study, 42 out of 60 patients (70%) (38 women and 4 men) had oral lesions, while 18 patients (30%) did not. The most common areas for the lesions were the buccal mucosa (26.1%) and the lips (14.2%). Out of the 42 patients with oral lesions, 12 (27.6%) patients had ulcers, 7 patients (16.6%) had erythema with a white centre, 5 patients (11 .8%) had only erythema and only one patient had white plaques (2.3%).

There was a significant relationship between the following factors and oral lesions: oral hygiene status (*p* < 0.02), the duration of the disease (*p* < 0.003), the number of pregnancies after the diagnosis of the disease (*p* < 0.027), medications used for SLE treatment other than corticosteroids (*p* < 0.008) and the daily use of corticosteroids(*p* < 0.046), without a significant difference between the doses groups, as *p* values for the three groups were *p* = 0.104, *p* = 0.213 and *p* = 0.412 respectively.

Conversely, age, gender, cigarette smoking and medications other than those used for SLE treatment were not significantly related to the presence of oral lesions (*p* value was greater than 0.05 in all subjects. Table 1 shows this data.

**Table 1:** Age, gender, cigarette smoking and medications other than those used for SLE treatment

Oral manifestation	Oral involvement	No oral involvement	Total	*p* value
Gender				0.396
**Male**	4	2		
**Female**	38	16		
Age				0.251
**Less than 18 years**	7	2		
**Between 18 and 45 years**	21	10		
**More than 45 years**	14	6		
Oral hygiene				0.014
**Good**	24	8		
**Medium–poor**	18	10		
Dental status				0.0541
**Dentulous**	29	8		
**Edentulous**	13	10		
Smoking				0.826
**Smoker**	18	7		
**Non-smoker**	24	11		
Disease duration				0.001
**Less than 3 months**		1	2	
**3–6 months**		3	9	
**6–12 months**		6	9	
**More than 12 months**		8	8	
Pregnancy				0.018
**Pregnant**	30	13		
**Not pregnant**	12	5		
Steroids				0.046
**Not taking**	0	0		
**Less than 7.5 mg**	10	10		
**7.5–60 mg**	16	4		
**More than 60 mg**	26	4		
Other SLE drugs	52	13		0.006

## Discussion

The total patient’s number was 62. Out of the total number, 42 (70%) had oral lesions, while (30%) had not. The majority of the patients were females. When searching the literature on the oral manifestations of SLE, we found several international studies dealing with the same issue. The 1973 Orman study showed the presence of oral lesions in 47 patients out of 182 [35]. Also, the results of the De Rossi study showed a frequency of oral injury ranging between 81.3 and 87.5%, and cases of coating and lingual inflammation were recorded [36]. Another study by Johnson and his assistants in 1984 on 51 patients demonstrated a prevalence of oral lesions in 51% of sample [37].

As for Katibi’s study, it showed similar results to our study in terms of the type of oral lesions and their pattern of its distribution, despite that their sample size was bigger (188 patients) [38]. Similarly, the types of lesions also agreed with Sabio’s study [39].

These differences in the results may be attributed to the difference in the studied race, the difference in the distribution of the lesions and their types and the small size of the sample, which did not find any correlation between gender, age, smoking and the use of medications. This correlation may appear with the presence of a larger number of patients [35–37, 39], as found by a reference analytical study of research publications dealing with this topic [39]. The lack of good dental care leads to an increased incidence of oral involvement, such as ulcers and infections, and the presence of good oral health care reduces these lesions [40, 41].

This study has shown that there is no correlation between the presence or absence of natural teeth, the installation of bridges and oral injury, and the frequency of oral lesions with the progression of the disease. This is due to the fact that most of the dermato-mucosal lesions manifest in the acute phase of the disease and decreases with the development of the illness, which is perhaps due to the remission. This matter has not been studied in our study and is considered one of its weaknesses, as we were unable to conduct the laboratory investigations necessary to complete the criteria for the remission or activeness of the disease. This was because lab tests at the hospital were only available at certain times, and patients had no financial abilities to perform the tests outside the hospital. Also, referring to different laboratories may lead to a mismatch of the results, and this will enter in other statistical equations [39, 42, 43]. Finally, oral lesions in our sample increased during pregnancy.

It is also very important to know that there are some types of oral lesion that could confuse us, like the lesions associated with Behcet’s disease, the disease which is common in some population in Middle Eastern regions. The diagnosis is based on the clinical manifestation and the lesion type and if these lesions are painful or not.

## Conclusion

Oral lesions are a common manifestation of SLE, especially on the buccal mucosa and lips, and the most common lesions are ulcers. Oral hygiene status, the duration of the disease, the number of pregnancies after the diagnosis of the disease, medications used for SLE treatment other than corticosteroids and the daily use of corticosteroids (without a significant difference between the doses groups) all showed a significant relation to the incidence of oral lesions in SLE patients. On the other hand, age, gender, cigarette smoking and medications other than those used for SLE treatment were not significantly related to the presence of oral lesions.

## Data Availability

All data generated or analysed during this study are included in this published article, and for any additional information, they are available from the corresponding author on reasonable request.
